# Pilot study of home-based delivery of HIV testing and counseling and contraceptive services to couples in Malawi

**DOI:** 10.1186/1471-2458-14-1309

**Published:** 2014-12-20

**Authors:** Stan Becker, Frank O Taulo, Michelle J Hindin, Effie K Chipeta, Dana Loll, Amy Tsui

**Affiliations:** Population, Family and Reproductive Health Department, Johns Hopkins School of Public Health, 615 N. Wolfe St., Baltimore, Md 21205 USA; Center for Reproductive Health, College of Medicine, University of Malawi, Mahatma Gandhi Road, P/Bag 360, Chichiri, Blantyre 3, Malawi

**Keywords:** Couples, Counseling and testing, Contraceptive services, Malawi, Home-based services

## Abstract

**Background:**

HIV counseling and testing for couples is an important component of HIV prevention strategies, particularly in Sub Saharan Africa. The purpose of this pilot study is to estimate the uptake of couple HIV counseling and testing (CHCT) and couple family planning (CFP) services in a single home visit in peri-urban Malawi and to assess related factors.

**Methods:**

This study involved offering CHCT and CFP services to couples in their homes; 180 couples were sampled from households in a peri-urban area of Blantyre. Baseline data were collected from both partners and follow-up data were collected one week later. A pair of male and female counselors approached each partner separately about HIV testing and counseling and contraceptive services and then, if both consented, CHCT and CFP services (pills, condoms and referrals for other methods) were given. Bivariate and multivariate logistic regression analyses were done to examine the relationship between individual partner characteristics and acceptance of the services. Selected behaviors reported pre- and post-intervention, particularly couple reports on contraceptive use and condom use at last sex, were also tested for differences.

**Results:**

89% of couples accepted at least one of the services (58% CHCT-only, 29% CHCT + CFP, 2% CFP-only). Among women, prior testing experience (p < 0.05), parity (p < 0.01), and emotional closeness to partner (p < 0.01) had significant bivariate associations with acceptance of at least one service. Reported condom use at last sex increased from 6% to 25% among couples receiving any intervention. First-ever HIV testing was delivered to 25 women and 69 men, resulting, respectively, in 4 and 11 newly detected infections.

**Conclusions:**

Home-based CHCT and CFP were very successful in this pilot study with high proportions of previously untested husbands and wives accepting CHCT and there were virtually no negative outcomes within one week. This study supports the need for further research and testing of home- and couple-based approaches to expand access to HCT and contraceptive services to prevent the undesired consequences of sexually transmitted infection and unintended pregnancy via unprotected sex.

## Background

Two common elements of reproduction and heterosexual HIV transmission are that they involve both men and women and they occur in the context of sexual partnerships. Furthermore, use of condoms can prevent both pregnancy and HIV transmission. Partnership contexts are critical to the explanation of pregnancy and HIV risk behaviors, and the dynamics of sexual partnerships need to be better understood. While some studies have shown that linking HIV to other reproductive health outcomes can increase the number of male clients reached, improve uptake of HIV counseling and testing (HCT), and condom use; the evidence is not always consistent [1, 2]. Experimental studies in Ethiopia and Turkey have shown higher rates of contraceptive acceptance and continuation when both husbands and wives are counseled, compared to only one partner [3, 4]. A review in 2010 of six high quality couples-based HIV intervention studies indicated that couples interventions were still in their infancy and should be conceptually grounded in an understanding of the structure and processes of couple relations that affect sexual risk behaviors, such as communication, power dynamics, intimacy, and fidelity [5]. The impetus for couple-based interventions has increased in recent years [6]. One reason for this is the belated recognition that in sub-Saharan Africa a large proportion of HIV infections occur within the context of stable relationships either as a result of previous infection or infidelity [7, 8]. Also, de Walque reported that in five African countries, two-thirds or more of couples with at least one HIV-infected partner were serodiscordant [9].

Formative research in Botswana comparing couple HCT (CHCT) to individual HCT finds that most participants preferred the former. However, participants warned that couples interventions must be carefully planned and implemented to avoid blame, misunderstanding, mistrust and violence [10]. Randomized and observational studies find that couples receiving CHCT show greater subsequent use of preventive measures, including condoms and nevirapine to prevent maternal to child transmission, than those receiving individual HCT [11–14] A CHCT-HCT comparison study in Lusaka and Kigali found reduced loss to follow-up for those receiving CHCT and there is greater cost-effectiveness for CHCT [15, 16].

With regard to home-based HIV counseling and testing (HBHCT), a recent review of 21 studies found that: a) it leads to much higher uptake than clinic-based testing (the average percentage for HBHCT was 83%); b) men are much more likely to be reached (average percentage of men was 47%), c) no harm was reported and d) many previously undiagnosed HIV cases are found [17]. In an island community of Malawi, Helleringer showed high uptake of HBHCT [18] and studies in Uganda have detailed further benefits. One study found HBHCT to be the most cost-effective per client tested as compared to stand alone HCT, hospital-based HCT and HCT for other members in households with an HIV-positive individual [19]. Another Ugandan study compared home and clinic-based testing and randomized household members on ART to these interventions. HBHCT was associated with lower HIV prevalence, higher uptake, and increased identification of HIV-infected persons than the clinic-based intervention [20]. A mixed methods evaluation of an HBHCT intervention in Uganda found it to be acceptable and effective--uptake of test results increased significantly as compared to clinic samples [21]. In two recent studies of HBHCT in Kenya and South Africa, about 45% and 14% of couples in the respective sites tested as couples [22, 23].

Two recent randomized controlled trials have also shown superior performance of HBHCT *vis. a vis.* couples testing. In Zambia 36 rural clusters were randomized to receive home vs. usual (clinic-based) testing services. In the HBHCT arm, 70% of couples tested and received results together while in the control arm the comparable figure was 51% [24]. In another study in rural Kwa-Zulu province South Africa, 16 clusters were randomized to receive HBHCT or usual clinic-based services. The results showed that 21% in the intervention arm received couple counseling compared to only 10% in the control arm [25]. In another approach to the same question, 300 women attending an antenatal clinic in Kenya were randomized to receive HBHCT or an invitation to the partner to come together for testing. The result was that 85% of couples tested in the HBHCT arm but only 36% in the “clinic-invitation” arm [26].

With regard to contraception, studies in Asia and Africa have demonstrated the benefits of home-based delivery of contraceptive services, particularly in rural areas. Under the label, “community-based distribution” (CBD), this home delivery was first tested in the 1960s. One of the best known CBD projects, the Bangladesh Matlab project, offered door-step delivery of contraceptive pills, condoms and child vaccinations. The project area, compared to the comparison area, showed large, statistically significant and enduring increases in uptake of contraceptive and other maternal and child health services [27, 28]. Other studies in Asia and Africa have demonstrated the benefits of contraceptive CBD [29–32]. A review showed that CBD models expand access to contraception, particularly to populations in isolated areas [33].

The primary aim of this study is to estimate the uptake of CHCT and couple family planning (CFP) services delivered to *couples* in their *homes* in a single visit. To our knowledge, this combined approach has not been tested.

## Methods

The study site is Mpemba, a peri-urban area of Blantyre, Malawi. The Blantyre region had an HIV prevalence of 22% among women and men ages 15–49 in 2004 [34]. Also 95% of married women are in monogamous relationships (97% of males; see below) [34]. Mpemba lies within the catchment area of Queen Elizabeth Central Hospital (QECH), one of the country’s main tertiary hospitals, that provides the full range of HIV testing, counseling and treatment services. Prior to the study, project staff met with community leaders to explain the purpose of the research. These meetings and a qualitative study in the same area confirmed that community members welcomed receiving home-based delivery of CHCT and CFP [35].

Three villages within Mpemba were selected and a household listing was undertaken in September 2009 to identify those with eligible couples. An eligible couple was defined as a man-woman pair married or in union with the woman aged 15 to 49 years and the man aged 15 years or older. Additionally, both partners had to co-reside in the household at least one day each week and claim the household as home. Polygamous men with co-resident wives were not included.

A study sample size of 180 couples was determined; this number provides estimates of proportions with a 95% confidence interval whose width (4*SE) is 0.15 (e.g., 50% ± 7.5%). Absent *a priori* data, we estimated that up to 50% of eligible couples would choose not to participate, requiring that at least 360 eligible couples be identified. We listed occupants of a total of 610 households in the three villages and identified 390 couples therein. After 198 households were visited, 180 couples were enrolled to participate in the study for a response rate of 91%. All eligible couples in one village and about a third of those in the two other villages were approached. If one member of a couple was not available, repeated attempts were made to reach the couple at a time where they were both available. The study protocol was approved by the Research Ethics Committee of the University of Malawi’s College of Medicine and the Committee on Human Research of the Johns Hopkins School of Public Health.

### Description of CHCT and CFP home-based services intervention

A pair of male and female counselors subsequently visited each couple. All counselors received intensive training in CHCT and CFP for five days, and each team had a qualified phlebotomist. After introducing themselves to both partners and providing an overview of the study, the counselors asked to meet privately with each partner and consented her/him to a baseline interview. The instrument included questions on marriage/union duration; schooling level; parity; current pregnancy; current use of contraception; desire for additional children; condom use at last sex with the partner; other sex partners in the last week; ever experience of physical violence from the partner; and ever tested for HIV. The instrument also included two attitudinal questions: “How emotionally close do you feel to your partner/spouse on a scale from 1 to 10, where 1 means no emotional closeness and 10 means very strong emotional closeness?” and “If you were to find out today that you/your partner were pregnant, how would you feel on a scale from 1 to 5, where a 1 means that you would be very unhappy to have a child now and a 5 means that you would be very happy to have a child now?”

After the baseline questionnaire, the woman’s counselor sought a private location and asked her consent to CHCT + CFP, CHCT-only or CFP-only. That counselor then used color-coded cards to discreetly relay the woman’s accepted intervention(s) to the male partner’s counselor in a second private location (often outside the back of the house). The man was offered whichever service(s) the woman had accepted. If the woman accepted neither, the man was not offered any of the services and the session ended. If the man declined the service(s) accepted by the woman, both were individually informed they were not eligible for the study and also given referral cards for Queen Elizabeth Central Hospital services. [This process was worked out in conjunction with the Malawi investigators and the Johns Hopkins Institutional Review Board to protect the women from coercion and potential violence to the maximum extent possible]. Without regard to which, if any, services were accepted, all couples received referrals to the same hospital for family planning services, HIV testing, and domestic violence counseling should such be desired. These procedures were followed to protect the woman from possible negative consequences.

For those who consented to both CHCT and CFP, the counselors reviewed the details of the interventions with each partner individually and then again as a couple, i.e., they would receive pre-test counseling together, individually have a rapid HIV test, and then receive CFP services and condoms in the 20–40 minutes while awaiting test results. These services included pills (brand Lo Femenol), condoms and resupply of Depo-Provera or referrals for new injection users and sterilization for nonpregnant women. Couples who chose not to have CHCT were offered the contraceptive services only.

The rapid HIV tests followed national standards; Determine and Unigold rapid tests were administered in parallel. Determine’s sensitivity on whole blood samples has been estimated at 100% and specificity at 96.2% and above [36]. Unigold has sensitivity estimated at 100% and specificity estimated at 99.7% [37]. A third rapid test, Ora-Quick, was available as a tie-breaker; however there were no ties.

Test results were first provided individually and confidentially. During post-test counseling each partner confirmed his/her willingness to disclose results to the partner. For those who consented to sharing results, the counselors brought the couple back together and provided the appropriate post-test counseling -- this included the importance of mutual support, staying healthy through good nutrition, practicing protective behaviors including condom use, and mitigating any risk of marital discord. A follow-up visit was scheduled for one week later.

If either partner changed his/her mind about disclosure, then after post-test counseling individually, the couple was brought together for generic HIV counseling covering the four possible scenarios: both HIV-positive, both HIV-negative, and discordance with either partner being HIV-positive. HIV-positive partners who disclosed were given transportation funds to attend QECH for further testing and disease staging. In couples with one or both partners HIV-positive, if counselors sensed a tense situation, they revisited the following morning to check on the couple’s well-being.

Counselors visited each couple a week later and at that time administered a brief follow-up questionnaire to each partner individually. Each was asked about a) sexual intercourse and condom use with the partner/spouse; b) sexual activity outside the partnership; c) current contraceptive use; d) physical violence from the partner; e) discussion with the partner about the HIV test and/or family planning counseling, and f) for HIV-positive persons, any follow-up visit to the referral clinic.

### Statistical analyses

Proportions of couples accepting the services were calculated. Means for interval variables and percentages for categorical variables were tabulated for couples in each of the acceptance groups and ANOVA and chi-square tests done to detect differences between groups. Factors associated with couple acceptance of CHCT and/or CFP were assessed using multinomial logistic regression with three groups – accepted CHCT only, accepted CHCT and CFP, and non-acceptors (as reference group). This method allows efficient comparison of each of the two acceptor groups with nonacceptors. Results are given as relative risks of being in the group (relative to being in the nonacceptor group) for a given level or value of the covariate [38]. We also examined couple concordance both pre and post-intervention on condom use at last sex and contraceptive use. We used McNemar’s chi-square tests for dependent samples since the same couples were interviewed at two time points [39]. For the follow-up data we attempted to minimize social desirability bias by only tabulating concordant reporting (e.g. both spouses had to report condom or contraceptive use). For the analyses Stata software was utilized [40]. We have used the traditional p-value of 0.05 as the cut-off for statistical significance.

## Results and discussion

**Figure 1:**
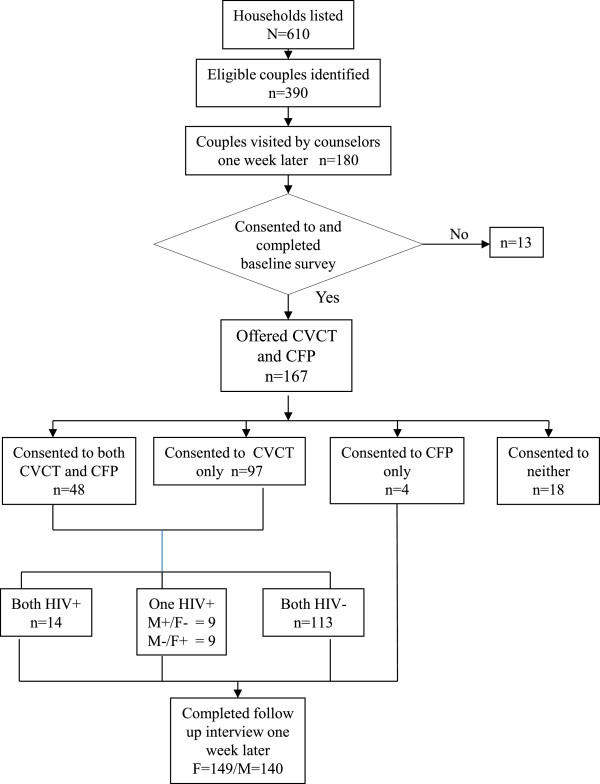
**Flow diagram for home-based delivery of couples VCT and FR service in Peri-Urban Malawi.**

Figure 1 is a flow diagram for the study. Of the 180 couples who agreed to participate at the initial listing visit, 13 subsequently declined to participate in the baseline survey. Of the 167 couples offered CHCT + CFP services, 48 (29%) accepted the combined service intervention; 97 (58%) consented to CHCT only; 4 (2%) to CFP only and 18 (11%) declined any intervention (17 women declined and one man declined after the wife had accepted CHCT). The study tested 145 couples for HIV, finding 14 (9.7%) concordant positive, 18 (12.4%) discordant (evenly divided between M+/F- and M-/F+), and 113 pairs concordant negative (77.9%). Among all the 149 couples accepting services, at least one member was successfully re-interviewed one week later--149 females and 140 males.

Table 1 presents background characteristics of the study couples as a whole and by accepted service. Considering all couples, men were about six years older than their partners, the percentages with any schooling were 88% for females and 90% for males, and the marriage was the first for about three quarters of partners. Male partners were significantly less likely than females to have been tested for HIV previously (55% and 81% respectively, p < 0.01). Negligible percentages reported having other sexual partners in the past week and reports of any physical violence ever from the partner were 20% for females and 8% for males. Over 65% of couples reported using contraception at baseline and three-quarters of them were using injectables.

**Table 1:** **Means and percentages of background characteristics of study couples by sex and intervention accepted**

Characteristic	Intervention accepted and sex (%) ^a^							
	All study couples n = 167		CHCT and CFP n = 48		CHCT only n = 97		None n = 18	
Means	Female	Male	Female	Male	Female	Male	Female	Male
Age (years)	28.3	34.8	27.5	33.6	29.0	35.7	25.3	31.3
Duration of union (years)	8.9	9.1	9.4	9.8	8.9	9.3	6.8	7.6
Number of live births	3.4*	3.4	3.5	3.4	3.5	3.5	1.8	2.3
Emotional closeness to partner score^b^	8.3*	8.6	8.5	8.7	8.4	8.6	6.7	8.1
Percentages								
In first union	77	72	81	73	71	71	94	78
With any schooling	88	90	90	94	87	90	94	89
Unhappy if discovered pregnant now	71	71	79	81	71	70	53	60
Using contraception	66	65	60	60	70	68	56	56
Reporting condom used at last coitus with partner	15	15	23	21	12	19	6	6
Reporting ever had HIV test	81*	55	88	46	80	56	61	67
Reporting other sex partner in last week	1	2	0	2	2	2	0	0
Reporting ever any violence from partner	20	8	15	6	24	10	17	0

^a^4 couples accepting CFP only are not shown separately; ^b^1 = no emotional closeness … 10 = very strong emotional closeness.

*p < 0.05 for test of hypotheses of equal percentages in each intervention group for a given sex.

Comparing across intervention groups by sex and based on ANOVA tests, emotional closeness and number of live births were significantly different for females in either intervention group versus the non-acceptance group (Table 1). There was also a pattern in reports of condom use at last sex with the highest for those in the CHCT + CFP group (23% female and 21% male), lower in the CHCT-only group, and lowest (6%) in the non-acceptor group.

The top left panel of Table 2 provides the unadjusted relative risk estimates for the association between background covariates and the likelihood of accepting either CHCT only or CHCT and CFP. Five covariates--age, prior HIV test, parity, emotional closeness and relative happiness/unhappiness if it was discovered that the wife is pregnant--had statistically significant bivariate associations with accepting CHCT or both services. (Also tested in simple multinomial models (for males and females separately) and found not significant at p < 0.10 level were: any schooling, condom use at last sex, current use of contraception and ever physically violent with partner.) Couples in which females had previously tested for HIV had the highest relative risks of accepting both interventions (RR = 4.45; 95% CI = 1.3,16.0). Relative risks of acceptance of CHCT or both interventions increase significantly with parity and with reported emotional closeness as reported by women. The right top panel of Table 2 shows odds ratio estimates for acceptance of either CHCT or CHCT and CFP together. As expected, the patterns are nearly the same. In the lower panel of Table 2 the multiple multinomial results are given with the female covariates from the top panel, except age was dropped because it was highly correlated with parity (r^2^ = 0.77). Female covariates were used because their relationships with the outcomes were stronger. (See top panel.) Relative risk estimates are only slightly attenuated from those seen in the single variable analyses.

**Table 2:** **Estimated relative risks for selected covariates from simple multinomial regression and multiple multinomial regression of couple acceptance of CHCT only or CHCT and CFP counseling and odds ratios from simple logistic regression for acceptance of either or both interventions by sex**

Covariate	Intervention accepted (reference = none) and sex for covariate studied					
	Accept CHCT only (n = 94)		Accept both CHCT and FP (n = 48)		Accept either or both (odds ratios from binomial logistic regression)	
**UNADJUSTED RELATIVE RISK RATIOS**					**UNADJUSTED ODDS RATIOS**	
	**Female**	**Male**	**Female**	**Male**	**Female**	**Male**
Age	1.08 [0.99, 1.16]	1.05 [0.99, 1.11]	1.05 [0.97, 1.14]	1.03 [0.97, 1.10]	1.07 [0.99, 1.15]	1.05 [0.99, 1.11]
Prior HIV test	2.61 [0.89, 7.63]	0.63 [0.22, 1.81]	**4.45 [1.24, 16.0]**	0.42 [0.14, 1.31]	3.16* [1.12, 8.93]	0.56 [0.20, 1.58]
Number of live born children	**1.69 [1.17, 2.43]**	1.29 [0.99, 1.70]	**1.68 [1.16, 2.43]**	1.27 [0.95, 1.68]	1.70* [1.19, 2.43]	1.29* [1.0, 1.69]
Emotional closeness	**1.39 [1.12, 1.72]**	1.17 [0.89, 1.55]	**1.45 [1.13, 1.86]**	1.24 [0.91, 1.70]	1.42* [1.15, 1.74]	1.20 [0.92, 1.57]
Unhappy^a^	0.39 [0.14, 1.09]	0.52 [0.19, 1.42]	0.33 [0.11, 1.0]	0.37 [0.12, 1.14]	0.38* [0.14, 1.02]	0.48 [0.18, 1.27]
**ADJUSTED RELATIVE RISK RATIOS** ^**b**^					**ADJUSTED ODDS RATIOS**	
Prior HIV test	2.07 [0.61, 7.03]		3.45 [0.84, 14.18]		2.40 [0.72, 7.99]	
Number of live births	**1.69 [1.12, 2.56]**		**1.67 [1.10, 2.56]**		**1.70 [1.13, 2.56]**	
Emotional closeness	**1.46 [1.15, 1.84]**		**1.59 [1.15, 1.98]**		**1.48 [1.17, 1.86]**	
Unhappy^a^	0.90 [0.27, 2.92]		0.77 [0.21, 2.76]		0.86 [0.27, 2.74]	

Note: Values in bold are significantly different from 1.0 at the p < 0.05 level.

^a^Reference is unhappy if found out the wife is pregnant now.

^b^Values for all covariates in the multiple multinomial model are from women.

The intervention delivered HCT services to many for the first time. Of 32 untested women and 76 untested men in the 167 couples at baseline, 78% of these women and 91% of the men received their first HIV test, with 4 women (16%) and 11 men (14%) learning their HIV-positive status; two of these were sero-concordant HIV-positive couples.

From re-interviews one week later, in about 60% of couples tested, both partners reported discussing last week’s HIV test (Table 3) with 12% saying it was for the first time. Among HIV-positive participants (n = 45), over half (57% of females and 55% of males) reported going to the clinic for follow-up care in the previous week (not shown). Concordant positive reports on both condom use at last sex in the week since the intervention and contraceptive use increased relative to baseline levels in both intervention groups (Table 4). Reported condom use at last sex increased from 6% to 25% among couples receiving any intervention. At the one week follow-up visit, no incidents of serious violence were reported among any of the couples.

**Table 3:** **Percentage of couples at one-week follow-up with positive concordant reports of specific behaviors since the counseling visit, by intervention accepted**

Behavior	Intervention accepted (%/N ^a^ )		
	All interventions ^b^	CHCT + CFP	CHCT only
Coitus in last week	74 (140)	67 (46)	48 (90)
Talked with partner about HIV testing	63 (136)	65 (46)	61 (90)
First time talked with partner about HIV testing	12 (85)	7 (30)	15 (55)
Talked with partner about FP counseling	41 (140)	57 (46)	33 (90)
First time talked with partner about FP	11 (57)	4 (26)	17 (30)

^a^The numbers in parentheses are sample sizes for the given percentages.

^b^Including 4 couples who accepted CFP only.

**Table 4:** **Percentage of couples with positive concordance on family planning use and condom use at last sex, pre and post intervention (one week later) by intervention accepted**

Measure/Acceptance group (n)	Pre	Post	Difference ^a^ (post-pre) (95% CI)
Using family planning			
All interventions (140)^b^	61	73	+12 (5,18)
CHCT only (90)	63	69	+6 (-1,12)
CHCT & CFP (46)	57	80	+24 (11,37)
Using condom at last sex^c^			
All interventions (102)	6	25	+19 (9,28)
CHCT only (71)	3	23	+20 (9,30)
CHCT & CFP (31)	13	29	+16 (0,35)

^a^This figure may be off by 1% from the difference of pre and post levels, due to rounding.

^b^Including 4 couples in family planning-only group.

^c^For the follow-up this question refers to coitus in the past week so only those who reported coitus are included and these couples are subset from all the couples responding in the baseline.

## Conclusions

This study was unique in offering both contraceptive and HIV counseling and testing to couples in their homes. Earlier studies have found that couple-based services are cost-effective, that integration of services is important and that delivery of services to the home is important; but this is the first to combine all three elements. We found that a large majority (83%) of couples in this area of peri-urban Malawi accepted HBHCT and/or family planning services, consistent with the literature cited earlier. CHCT/CFP interventions led to increases in couple communication (Table 3) consistent with what has been found in other settings [41]. The low acceptance of the CFP component may be because contraceptive use was already high (66%, see Table 1) due to an active private family planning program in the area.

The formative study that preceded this quantitative one [35] identified some of the potential social benefits and costs of this home- and couple-based intervention. These included the convenience and lower cost of combined delivery of HCT and family planning to couples. Also the intervention was expected to lead to higher levels of testing that would enable more partners to know their status and for sero-discordant couples to have protected sex. Female partners were particularly concerned about their vulnerability to infection and unplanned pregnancy in the absence of their male partners’ involvement. The formative study included focus groups with couples, and investigators were surprised at the degree to which covert use of contraception was disclosed by wives. Concern about the consequences of deviating from gender and social norms remains important for this intervention design and while no adverse outcomes, such as intimate partner violence, were observed for females, the small sample size prevents generalization.

This pilot study has some limitations. First, the sample size is small so only large differences could be detected as statistically significant; however, as planned we could estimate that the percentage accepting one or both services in this population lies in the interval 83% ± 5% with 95% confidence. Second, there was no control group, as this was designed as a pilot study to assess feasibility. However, first-time testing and newly detected HIV infections showed that in the absence of the intervention, 11 men and 4 women would not have known they were infected. Thus the intervention was quite effective in reaching untested sero-positive individuals. From the Malawi DHS that preceded this study (i.e. DHS of 2004), among 1850 couples in the national sample, although over 98% of both husbands and wives had heard of HIV/AIDS, only 15% of wives and 17% of husbands had ever been tested. Third, social desirability bias could distort the findings, particularly since the same counselors visited one week later to administer the post-intervention questionnaire. We adopted this approach because the counselors had established some rapport with participants the previous week and, in the interests of human subjects’ protection, they were better positioned to deal with any problems arising in the couple. The risk of social desirability bias is mitigated somewhat since the follow-up responses come from interviews with each partner independently and only concordant reports are presented in the results. Finally, the follow-up period was only one week; changes that occurred in the longer term were not observed.

HIV counseling and testing is one pillar of strategies to prevent transmission. Despite the availability of free testing at health facilities close to the study area, a large percentage of male partners had never been tested. The intervention delivered HCT services accepted by 78% and 91% of the untested female and male partners respectively, and reported use of condoms also increased substantially after the counseling visit.

The likelihood of a couple’s acceptance of services was positively and significantly associated with a woman’s number of live births, reported emotional closeness to her partner, and prior HIV-testing. Fewer male covariates were significant in predicting acceptance, possibly because males were only offered the services that their partners first accepted. In 17 of the 18 cases it was the woman who declined services.

Similarly, it was women’s reported emotional closeness (not men’s) that was a significant predictor of acceptance of the interventions. A crosstabulation of the husband’s report and wife’s report showed that in 80% of couples the reports of the two spouses were within 2 points of each other (i.e. between -2 and +2 on a scale of differences of -9 to 9) but from the regression results, the wife’s report was a more valid predictor of couple uptake of the services. The husbands appear to be less attuned to the state of emotional closeness in the couple. The implication for programs is that in CVCT interventions the wife be approached for her consent first.

HBCHCT and HBCFP warrant further study and any scale-up will invoke operational considerations. First, the combined approach should be tested using an experimental design. Second, the costs and cost-effectiveness of integrated home-delivery services should be tracked and assessed. The level of start-up costs will depend not only on the country setting but also on client load for community-level workers. Third, protocols need to be developed for sites where polygamous men have co-resident wives. Fourth, this pilot study had stopping rules in place in case of serious violence, but no incidents were reported in the week post-intervention.

Semrau and colleagues have also reported that couple counseling in Zambia did not increase the incidence of adverse events over individual counseling [42]. Though earlier studies [43, 44] documented violence associated with HIV-status disclosure of HIV-positive women to their partners, a literature review of CHCT interventions indicated no additional risk associated with disclosing results to a partner [7]. With respect to relationship characteristics such as trust, intimacy and so on, we were unable to assess the effects of the intervention. However, a majority of couples did report that they talked about HIV testing in the week after the intervention.

The findings from this pilot study support further research and testing of home- and couple-based approaches to prevent the undesired consequences of unintended pregnancy and sexually transmitted infection through unprotected sex in Malawi and elsewhere in Africa. The approach enabled first-time testing for significant proportions of men and women, detected new infections among men and women, and facilitated couple-level discussion around sexual and reproductive risk behaviors.

## Authors’ information

SB has done research on couples and reproductive health for over 15 years. FT, as former director of the Center for Reproductive Health at the University of Malawi, managed the project directly. MH has worked on women’s and reproductive health concerns in many countries. AT, as director of the Gates Institute for Population and Reproductive Health, encouraged and spearheaded studies such as this one, in many countries.

## Abbreviations

CBD: Community-based distribution
CHCT: Couples HIV counseling and testing
CFP: Couples family planning
HBCFP: Home-based couples family planning
HBHCT: Home-based HIV counseling and testing
HCT: HIV counseling and testing
QECH: Queen Elizabeth Central Hospital.
